# Assessment of acceptability of the COVID-19 vaccine based on the health belief model among Malaysians-A qualitative approach

**DOI:** 10.1371/journal.pone.0269059

**Published:** 2022-06-14

**Authors:** Mohd Dzulkhairi Mohd Rani, Nurul Azmawati Mohamed, Hana Maizuliana Solehan, Muslimah Ithnin, Abd Rasyid Ariffien, Ilina Isahak

**Affiliations:** Faculty of Medicine and Health Sciences, Universiti Sains Islam Malaysia, Nilai, Negeri Sembilan, Malaysia; Ondokuz Mayis University Faculty of Medicine: Ondokuz Mayis Universitesi Tip Fakultesi, TURKEY

## Abstract

**Introduction:**

Several countries have started mass vaccination programs to halt the spread of the COVID-19 pandemic. With an R naught value of 2 to 3, about 70% of the population needs to be immunized to achieve herd immunity. This study aimed to investigate the reasons for acceptance or refusal of COVID-19 vaccines among the Malaysian population.

**Methodology:**

An exploratory, descriptive qualitative design was performed. The cross-sectional survey used a non-probability convenient sampling technique to recruit the respondents, who were required to answer an open-ended question: Either "If you are willing to get the vaccine, please state your reason" or "If you are not willing to get vaccinated, please state your reason." The survey also included questions on demography such as age, gender, and place of residence. According to the Health Belief Model, the data was transcribed, translated, and analyzed: perceived susceptibility, perceived severity, perceived barrier, and cues for action.

**Results:**

A total of 1091 respondents who completed the online survey comprised 685 (62.8%) females, 406 (37.2%) males, with a mean age of 38.16 (SD = 16.44). The majority (81.1%) were willing to get vaccinated. Thematic analysis showed that most respondents perceived that the vaccine is safe, effective, protective and will provide herd immunity. Barriers to vaccination include unknown long-term side effects, rapid vaccine production, inadequate information and concerns regarding *halal* status. Cues to vaccination included individual desire, social responsibility, economic concerns and wait-and-see behavior.

**Conclusions:**

The public should be well informed about the vaccine, its efficacy, side effects, and halal status to increase vaccine acceptability and achieve herd immunity.

## Introduction

The battle against COVID-19 is getting more intense as several countries have started their mass vaccination programs for COVID-19. The United Kingdom became the first country to vaccinate people with a fully tested vaccine from Pfizer-BioNTech [1]. As of January 2021, Malaysia is also preparing for a similar program, subject to approval from the National Pharmaceutical Regulatory Agency [2]. Vaccination speeds up the journey towards achieving herd immunity. Theoretically, the higher the R naught of an infection, the higher proportion of the population must be immune in order to decrease transmission. For COVID-19, most studies estimated that the R naught of SARS-CoV-2 was in the range of 2 to 3 [3]. Therefore, the herd immunity threshold would be expected to range between 50% and 67% [4]. This means that in order to achieve herd immunity, about 70% of the population should be immunized either through naturally acquired infection or vaccination.

Even though Moderna and Pfizer-BioNTech vaccines were highly effective and relatively safe, some people were still skeptical and refused vaccination [5]. The introduction of new vaccines in the market may be politically and economically complicated due to refusal or hesitancy to accept the vaccine, which may hinder the government’s aspiration to achieve herd immunity that can potentially end the pandemic [6, 7]. Although the insights and opinions of various stakeholders, such as policymakers and medical specialists, may to some extent affect the adoption of the vaccine, the most important factor for the successful adoption of any vaccination program is the acceptance of the public [8, 9]. Studies investigating the factors influencing the public’s preference for COVID-19 vaccine acceptance are currently fragmented and limited to mainly quantitative results [10, 11]. A study done amongst parents showed that parents’ willingness for their children to get COVID-19 vaccination was low but the associated influencig factors were not further explored [12].

Because qualitative research deals with human experiences, the complexities regarding COVID-19 and vaccine acceptance have provided challenges and opportunities for these multitudinous research studies [13, 14]. For instance, a study among adolescent revealed that, those who were vaccine hesitant had greater indicators of social deprivation and felt a lack of community cohesion [15]. The research question used to guide this exploration was: What are the health beliefs related to the acceptability of the COVID-19 vaccine among Malaysian adults?

Theoretical models of health beliefs and risk perception are important tools for understanding the factors that influence decision-making by determining what motivates and discourages people to engage in health-related behavior. This includes employing behavior change theories to provide a framework for comprehending vaccination hesitancy [16]. The Health Belief Model (HBM), the Theory of Planned Behavior, the Risk Perception Attitude, the Triandis Model of Interpersonal Behavior, and the COM-B (capability-opportunity-motivation-behavior) model are among the commonly used theories to predict vaccination uptake [16, 17]. The review also reported that the HBM was successful in predicting vaccination uptake and distinguishing perceptions of the vaccinated from non-vaccinated individuals in the study population.

This study aimed to investigate the reasons for accepting or refusing future COVID-19 vaccines among Malaysians using an exploratory, descriptive qualitative design. Findings from this paper are intended to generate discussion for much-needed solutions of COVID-19 by understanding the acceptance factors for a possible vaccine using the Health-Belief Model (HBM) [18].

## Materials and methods

### Study design and sampling technique

We conducted a cross-sectional survey among Malaysians aged more than 18 years. Non-probability convenient sampling technique was applied to recruit the respondents required to fill up a questionnaire. The inclusion criteria were social media users and having access to an internet connection to fill up the online questionnaire.

### Study instrument and setting

Two open-ended questions were designed to obtain information about acceptance of a new COVID-19 vaccination. The text of the questions read as "If you are willing to get the vaccine, please state your reason" and "If you are not willing to get vaccinated, please state your reason." The survey also included questions on demographic information such as age, gender, and state of residence. The questionnaire was developed in both English and Malay language, using Google form. It was then entered into an online survey system. A link to the electronic questionnaire was shared with respondents across Malaysia using social media platforms, specifically WhatsApp, Telegram and Facebook. We also requested our social media networks to share the electronic questionnaire with their networks. This was done to facilitate the recruitment of more respondents. The data was collected and analyzed daily, and saturation was defined as no new themes were generated from the responses [19]. After 15 days of data collection from 2 December until 17 December 2020, the link was closed.

### Theoretical framework

The Health Belief Model provided the theoretical framework to explore the acceptability of COVID-19 vaccination among Malaysians. The Health Belief Model constructs of interest were perceived susceptibility, perceived severity, perceived benefits, perceived barriers, and cues to action. Individuals who believe they are susceptible to the disease have perceived susceptibility. This could involve a person expressing concern about acquiring the infection; as a result, they understand that vaccination protects them from infectious COVID-19 diseases by stimulating the immune system to develop antibodies. Perceived severity refers to people’s beliefs about the significance of a particular health problem resulting from getting or not getting vaccinated, which can differ from person to person. Then, perceived benefits and barriers act as a catalyst for action, resulting in COVID-19 vaccination acceptance. Finally, a cue for action is used. This when a factor serves as a cue, or a trigger, to having the desired appears to be required, which is acceptance or reluctance to vaccination [20].

### Data analysis

The recorded interview data were verbatim transcribed into a Word document. The researchers were familiar with the complete data set by repeating and actively reading the interview transcripts. The researcher then produced preliminary codes from the transcripts. The research team then evaluated and compiled the transcripts based on the Health Belief Model themes. (19,21) The researchers independently studied the transcripts before examining the theme for this early familiarisation stage, initial coding, and theme generation. The information was coded and entered into a Microsoft Excel spreadsheet. The research team then defines and refines the theme. Transcripts of similar data were grouped to form sub-themes, which consensus by all authors then approved. Finally, the study includes writing up the final analysis and presenting the findings via narrative reporting by themes, sub-themes, and quotes. The back-to-back translation was carried out for Malay responses.

### Ethical approval

The study was conducted according to the guidelines of the Declaration of Helsinki, and approved by the Institutional Review Board (or Ethics Committee) of Research and Ethics Committee, Universiti Sains Islam Malaysia (USIM/JKEP/2021-126). The subjects consented to participate in this survey by agreeing to complete and submit the questionnaire.

## Results

**Fig 1:**
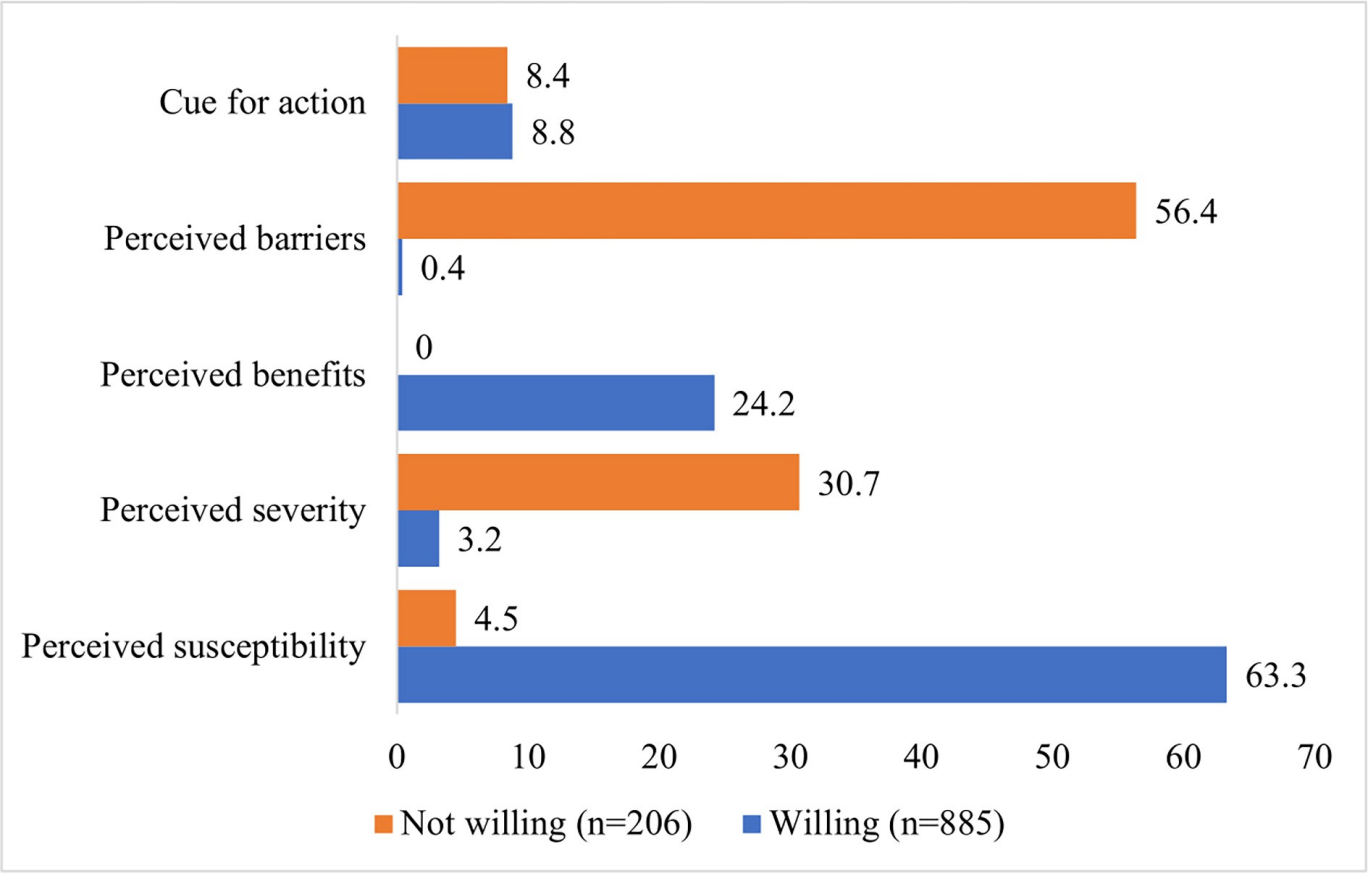
The percentages of acceptability to get COVID-19 vaccine based on the health belief model.

A total of 1091 respondents completed and submitted the informed consent and the questionnaire electronically via the online survey. Of all the respondents, 685 (62.8%) were females, and 406 (37.2%) were males. The mean age was 38.16 (SD = 16.44) and ranged from 18–89 years old. The majority of the respondents were from the state of Selangor (38.1%), followed by Kuala Lumpur (15.5%), Johor (7.9%) and Kelantan (6.5%). In response to the open-ended questions, most respondents (81.1%) were willing to get vaccinated, whereas 18.9% were not willing to be vaccinated. Fig 1 shows the percentages of acceptability to getting the COVID-19 vaccine based on the HBM. Most respondents were willing to get vaccinated against COVID-19 as they had a perceived susceptibility (63.3%). While among those who were not willing to get vaccinated, majorities had perceived barriers (56.4%).

Table 1 summarizes the themes and sub-themes in terms of the Health Belief Model’s core concepts obtained from this survey.

**Table 1:** Summary of themes and sub-themes in terms of the Health Belief Model.

HBM Concept	Themes	Sub-themes
Perceived susceptibility	Knowledge and perception of vaccination	1. Prevention is better than cure 2. Immunity 3. Negative perception on COVID-19
Perceived severity	Health effects of SARS-CoV-2 infections and vaccines	1. The vulnerable group likely to get infections 2. Susceptible to vaccine side effects
Perceived benefits	Benefits of vaccination	1. Vaccines are safe and effective 2. Continue normal lifestyle as before COVID-19 pandemic
Perceived barriers	Barriers to getting vaccinated	1. Disbelief in new vaccines and new technology used 2. Lack of information about vaccine 3. Limited clinical trial data 4. Type of vaccine 5. COVID-19 mutation and multi-strain 6. *Halal* status 7. Global media 8. Stigma and skepticism on the vaccine agenda 9. Health self-belief 10. Belief in available treatment 11. Follow the current regulation and standard operating procedure (SOP) set by the government 12. Cost of COVID-19 vaccines
Cues to action	Cues for taking the vaccine	1. Individual desire 2. Social responsibility 3. Belief in an authorized health organization 4. Economic concerns 5. Not the anti-vaccine group 6. Priority for high-risk groups 7. Wait-and-see

### Theme 1: Knowledge and perception of vaccination

Respondents stated that the use of vaccines to prevent infectious diseases has previously been proven to reduce the rate of disease occurrence. Vaccination is also considered an effort to prevent infection as they stated that taking the vaccine is better than curing the diseases. Those who were willing to take the vaccine believed that the vaccines could prevent the symptoms of COVID-19. Thus, the respondents believed that taking the vaccines will prevent them from getting COVID-19 infection.

*Vaccines are part of our effort to avoid infection, and they are a preventative method that has been proven by trusted clinical studies. (Willing, F, 23 y.o.)*

*Prevention is better than cure. To prevent further harm, vaccines are essential. (Willing, F, 20 y.o.)*

Immunity is the body’s ability to defend itself from ’foreign bodies.’ The respondents who were willing to get vaccinated believed that their bodies would build immunity through vaccination, thus preventing them from getting infected by the SARS-CoV-2 virus. The respondents also thought that through vaccination, they would also be able to protect not only themselves but also their family, including the elderly, and those who are immunosuppressed. Vaccination can help in achieving herd immunity. Respondents also believed that protecting oneself from disease is one of the demands of Islamic practices. Thus vaccination will confer immunity which is one way of disease prevention.

*I believe in herd immunity so that I can protect myself and other immunosuppressed in the community. (Willing, F, 23 y.o.)*

*Because protecting oneself from disease is one of the demands of Islam, so I strongly agree to take it. (Willing, F, 20 y.o.)*

As there were myths related to COVID-19 circulating in the media and the community, there was a negative perception of COVID-19. A respondent stated that he was unwilling to get vaccinated as he did not acknowledge the existence of the COVID-19 virus.

*I do not believe in the COVID-19 virus. (Unwilling, M, 28 y.o.)*

### Theme 2: Effects of SARS-CoV-2 infections and vaccines

Reports show high negative consequences of SARS-CoV-2 infection, including loss of life, particularly in older individuals and those with pre-existing comorbidities. Therefore, aged and high-risk respondents see themselves as a vulnerable group. Thus, they stated that they were ready to take the COVID-19 vaccine because taking the vaccine can prevent infection and reduce the risk of complications or death from the infection. For individuals working in high-risk fields such as front liners and frequent travelers, they were also willing to get vaccinated as this will give them more protection and allow them to work and travel with peace of mind.

*A person with comorbidity has a high risk of infection and its complications. (Willing, F, 63 y.o.)*

*I am old. I want to be protected. I do not want to be a burden in case I get the disease and suffer in silence. (Willing, M, 75 y.o.)*

*My job requires me to travel around the world. (Willing, F, 54 y.o.)*

*As a health worker seeing patients daily. (Willing, M, 68 y.o.)*

Respondents who were unwilling to get COVID-19 vaccination stated that pharmaceutical companies newly produced the vaccine soon after the global pandemic in March 2020. It is too early to take the vaccines as the vaccines may have unwanted short and long-term side effects, might be unstable, not proven 100% legit, and reported death cases during vaccine clinical trials among the volunteers. The new technology with genetic material used in vaccine development also made them unwilling to get vaccinated as the respondents were worried about the unknown side effects of the vaccines.

*It has not reached like one year above after the vaccine was created. It is too early to believe that the vaccine does not have side effects and is safe for everyone, especially the elderly and infants. (Not willing, F, 27 y.o.)*

*Not enough time to find side effects, especially long-term, because of new technology with genetic material. (Not willing, F, 68 y.o.)*

*I do not want to be among the guinea pigs. Let it be used for 2–5 years and proven no cray side effects; then I think it is ok to get it. (Not willing, F, 35 y.o.)*

### Theme 3: Benefits of vaccination

For respondents who are willing to take the vaccines, they believe that the vaccines will benefit them. For them, taking the vaccine is the correct way to control the COVID-19 pandemic. Vaccination is the right thing to do, as they are confident about the vaccine’s effectiveness, and it has a very low risk of side effects.

*The only way to truly control the COVID-19 pandemic. (Willing, M, 58 y.o.)*

*Confident in the effectiveness of vaccination and the very low risk of side effects (Willing, M, 49 y.o.)*

After the WHO announced the pandemic, the government regulated new rules and standard operating procedures to break the chain of transmission of COVID-19 in the community. Among the respondents, they believed that once they get vaccinated against COVID-19, they will be able to go back to and continue the normal lifestyle. The vaccination will benefit them in providing protection; thus, they will continue their normal routine, no more quarantine, and freedom to move, travel, and study normally at the university.

*I would like to protect myself and my loved ones from this disease so we can get to the normal environment again, doing daily routines without concern & travel with them. (Willing, F, 24 y.o.)*

*(I want to) quickly go back to normal life, to study normally at the university, everything (to go back to) normal. (Willing, M, 21 y.o.)*

### Theme 4: Barriers to getting vaccinated

Barriers to receiving vaccines emerged as another significant sub-theme revealing an overarching lack of acceptance to COVID-19 vaccines. Inherent in this category was the disbelief of the new vaccines and new technology used, lack of information about the vaccine, limited available clinical trial data, type of vaccine, concern on COVID-19 mutation and emergence of multi-strains, the *halal* status, negative global media reports, stigma and skepticism of the vaccine agenda, individual health self-beliefs, and belief in available treatment, preference to follow the current regulation and SOP set by the government and the cost of COVID-19 vaccines.

For some of the respondents who were unwilling to get vaccinated, in their opinion, the vaccination development was made in a rush. The safety of the new mRNA technology has not been proven, and it might alter human DNA. This requires further testing and data of results of clinical trials conducted on larger samples.

*Too little time has been done for testing, test samples too small, virus mutation and all these things just feels rushed. mRNA is a new method that has not been proven nor disproved. (Not willing, M, 38 y.o.)*

*Because it was produced too quickly and it can disrupt the receiver’s DNA. (Not willing, F, 60 y.o.)*

Another barrier in acceptance to COVID-19 vaccination was the lack of information about the vaccines. Many respondents commented that they were not well informed regarding the content of the vaccines and the efficacy and safety issues. Some reports make them more doubtful about the COVD-19 vaccines. Assurance was needed in terms of who would be responsible if the vaccine fails and who will compensate for any loss.

*Many reliable reports cast doubts on the safety rushed development vaccine. (Not willing, M, 59 y.o.)*

*Details of vaccine studies and its mechanics with our body cells mass must be made available, outcomes if vaccinations must be thoroughly discussed by the experts, peers and groups, information must be readily available from experts groups and I must be well informed on what it is all about. Whether it is inactivated or activated antigens and how successful it is expected to do its jobs. It should not be a case like any on-site terms and conditions where if you decline, you will have no access to social interactions. I have to be thoroughly informed on its real efficacy. I want to know who will be responsible if it is a failed product and how should my well-being be compensated by whom. I need reasonable assurance before I make the decision. (Not willing, M, 68 y.o.)*

*Disagree because we do not yet know the extent of the effectiveness of the vaccine to protect the body from COVID-19 infection and immunity or the risk of creating other diseases caused by individuals receiving the COVID-19 vaccine. (Not willing, F, 21 y.o.)*

According to the respondents, the development of the COVID-19 vaccine had the shortest period of vaccine development in history. Thus, they were unwilling to get vaccinated. Besides, they were not confident to take COVID-19 vaccines as there are limited clinical data with preliminary clinical trials, no longitudinal studies, no large scale or prolonged history of efficacy or reliability. Also the long-term effects on different people are still unknown, including among different age groups and Malaysian citizens. The long-term safety data are also not available.

*Not enough trial results to prove effectiveness. Vaccines may have been rushed to complete. (Not willing, F, 27 y.o.)*

*I do not know the effectiveness and side effects for the Malaysian community itself, also for different ages and backgrounds of different injection recipients. Too many parameters cannot be determined because the time to develop this vaccine is seen as too short. (Not willing, F, 27 y,o.)*

*I do not have confidence that the vaccine is safe, (I am) afraid of the effects a year or two later… making the virus more virulent. (Not willing, F, 21 y.o.)*

Another perceived barrier in accepting the COVID-19 vaccines was the lack of trust in the vaccine producer company or country of origin. According to them, the process of vaccine development by the vaccine development companies was not transparent. Some respondents questioned the reliability and capability of China and Russia in producing vaccines. They have more confidence in vaccines produced by the United States of America.

*Do not trust the countries & companies producing the vaccine! The process is not transparent & the regulatory authorities have been compromised! (Not willing, M, 72 y.o.)*

*Current vaccines only reduce the severity of COVID-19 symptoms, not protect against the pandemic; plus am not comfortable with genetic coding & that cells of aborted fetus used in Pfizer vaccines. (Not willing, F, 75 y.o.)*

*Provided that the vaccine is not made in China or Russia, compared to the United States, both countries are known not to conduct proper clinical trials. CCP in China is the cause of the spread of COVID-19 (that is why China is) unreliable. (Not willing, M, 39 y.o.)*

For some respondents, accepting the COVID-19 vaccine would depend on the type of the vaccine used. They stated that they would be willing to get vaccinated using the conventional type of vaccine (inactivated virus) rather than the new mRNA vaccines. For them, mRNA is a new type of vaccine. Thus, it may have a side effect as no longitudinal research study has been conducted.

*Only deactivated vaccine for protection against COVID-19. (Not willing, M, 63 y.o.)*

*Because long-term research has not been done… Additionally, this is the first mRNA to be used in humans… . What will happen if the body fails to produce antibodies, but the body itself has produced viral proteins through viral mRNAs that give instructions for the production of viral proteins in the human body? (Not willing, F, 41 y.o.)*

COVID-19 mutation and variants were another perceived barrier in acceptance to the vaccines. Virus mutations generate genetic diversity, subject to adverse selection actions and random genetic drift. Some respondents raised concern over the rapidly mutating COVID-19 virus.

*This corona outbreak only started late last year, while the new vaccines are produced within a few months, to produce a quality vaccine will often take years because of many experiments, side effects, changes and acceptance of a vaccine against the body, changes in the acceptance of the anti-vaccines in executing defenses to fight the virus itself. There is a lot to investigate, to think about… And from what I know, the virus itself mutates… while the vaccine is made according to the previous, current situation… Meanwhile, the mutation of the virus is often variable. I fear that if it is inappropriate, it will cause more harmful side effects in the long run. (Not willing, F, 40 y.o.)*

*If confident that there is no evolution of the virus, and this vaccine is able to deal with COVID-19. Why not? (Willing, F, 22 y.o)*

*Halal* is an Arabic term that means lawful or permitted. Among respondents, the *halal* status plays a vital role in their acceptance of the COVID-19 vaccine. For them, information on *halal* status is necessary to give them the confidence to take this vaccine. Those who were unwilling to take the vaccine believed that the available vaccine is non-*halal*, thus contributing to barriers to getting vaccinated. Several respondents assumed that vaccines contained non-*halal* ingredients, for example, fetal cells and porcine materials.

*Contains content that has impermissible elements, such as fetuses and pigs. (Not willing, M, 33 y.o.)*

*The halal status is not certain… the media always repeat the benefits of vaccines… without an in-depth examination… halal is very important for Muslims. (Not willing, F, 42 y.o.)*

In today’s world, various information and news can be obtained through the media and the internet. Some respondents received negative news and information regarding the COVID-19 vaccines’ subsequent adverse effects during the clinical trials from the internet and global channels. This contributed to their unwillingness to receive the vaccines.

*Not ready to receive the COVID-19 vaccine because there is no valid certification from any other country about the effectiveness of this vaccine. In fact, there are countries whose citizens have died from the COVID-19 vaccine. (Unwilling, F, 21 y.o.)*

*Suddenly there is news about six deaths due to "COVID-19 vaccine"… Ha, is that it? Forgive me if I have forgotten… . And why I do not agree is because I wonder if this is one of the Illuminati plans? An evil plan that seeks to wipe out some of the world’s population? Haa… that is one of the reasons why I do not agree … (Unwilling, M, 19 y.o.)*

The sub-theme of social stigma and skepticism of the vaccine agenda also emerged as a major barrier. Some respondents believed that certain parties had manipulated the purpose of vaccination. They were afraid of a hidden agenda beyond COVID-19 and the vaccines given.

*Lack of confidence in the materials used. I am worried about having side effects or having other agendas that we do not know because it was created by Westerners/non-Muslims. (Not willing, F, 21 y.o.)*

*The C19 virus is not so dangerous that it is necessary to vaccinate. (We are) exposed to manipulation by interested as well as malicious parties. (Not willing, M, 45 y.o.)*

Respondents also stated to have health self-belief. Some healthy respondents, particularly those young and without chronic diseases, were unwilling to get vaccinated since they believed that their body would mount adequate immune response during actual infection. Another belief is that they were always taking care of their health. Thus vaccination against the COVID-19 vaccine is unnecessary.

*COVID-19 attacks people who are weak in antibodies due to age and pre-existing diseases; why should it involve individuals who already have the ability to produce their antibodies? (Not willing, M, 40 y.o.)*

*I always take care of my health & I do not think it is necessary. (Not willing, F, 62 y.o.)*

Most of the cases of COVID-19 infection generally experienced mild illness and symptoms and is a self-limiting infection. Thus, some of the respondents who were unwilling to get vaccinated believed that the vaccines were not needed. For them, the vaccines were not proven to be effective and may also bring side effects. Thus, they preferred to use currently available treatment in treating the COVID-19 infection.

*For hospital treatment, no medication is given to patients if no symptoms are present. They are only placed in quarantine. And Alhamdulillah, they recovered. So why do you need to take this corona vaccine? (Not willing, F, 30 y.o.)*

*COVID-19 is just a common cold fever (so) why need a vaccine that is not sure to cure? And it may be harmful. (Not willing, F, 40 y.o.)*

For respondents who were unwilling to take vaccines, they stated that taking the vaccine is a risky behavior. The Malaysian government had released rules and regulations to be obeyed by Malaysian citizens to break the COVID-19 chain of transmission. Thus, the respondents said that they preferred to practice preventive measures as stated by the government, stay at home, and avoid gatherings or crowded places to protect themselves from COVID-19.

*Long-term adverse effects will be lethal. Let the front liners and Parliament M.P.s take it first. I would just stick to the new norm (wearing a mask, nonsense physical distancing). (Not willing, M, 23 y.o.)*

*Not sufficient time for trials to be detailed & all side effects to be recognized. This is a fast-track vaccine. I am 67, and I suffer side effects from many meds & antibiotics as it is. It would be too risky for me. I have been limiting my outings to a minimal, avoiding big groups & get-togethers & will continue to do so for my protection. (Not willing, F, 67 y.o.)*

Finally, the sub-theme of the cost of the COVID-19 vaccine emerged as a major barrier. Vaccine acceptance depends on the cost and availability of the vaccine.

*Willing to get if the vaccine is not too expensive. (Willing, F, 45 y.o.)*

*Depending on the price of the vaccine and its effectiveness. (Not willing, F, 43 y.o.)*

### Theme 5: Cues for taking the vaccine

The cues for taking vaccines were sub-categorized into individual desire, social responsibility, belief in an authorized health organization, economic concerns, not the anti-vaccine individual, prioritizing the high-risk group, and waiting and seeing behavior. Among respondents, vaccines against COVID-19 are essential to protect themselves from getting infected. They state that it is their desire to get vaccinated as they would do anything to find a cure, be protected, feel safer, and be in good health.

*I will do anything for good health. (Willing, F, 56 y.o.)*

*An action that should be taken as one of the important Fiqh which is saving lives. (Willing, M, 28 y.o.)*

If most of the population is immune to an infectious disease, this provides indirect protection for those who are not resistant to the infection or herd immunity. Among the respondents who are willing to be vaccinated, they considered vaccination as part of their social responsibility to contribute to herd immunity. By doing so, they can protect themselves and other people in the community. They believed that vaccination is the best way to break the SARS-CoV-2 chain of transmission in the community after compliance with the government’s SOP.

*This is one of the ways for us as citizens/society to play our social responsibility in fighting the spread of* COVID-19 *domestically and internationally*, *as proven in the control of infectious diseases before*. *(Willing*, *F*, *38 y*.*o*.*)*

*As a protective measure to yourself and others*. *Although I do not yet know the effectiveness of this* COVID-19 *vaccine*, *but I believe in the MOH who strives to do the best for Malaysians*. *(Willing*, *F*, *30 y*.*o*.*)*

The respondents’ belief in authorized health organizations is the cue for taking COVID-19 vaccines. They have full confidence that the relevant authorities had done their best before approving the vaccines. The authorized health organizations include the World Health Organization (WHO), U.S. Food and Drug Administration (FDA) and the Ministry of Health Malaysia. Additionally, for the *halal* status, they trust the wisdom of the religious authority.

*Assumed vaccine got approval from the authorized Health Body and WHO and FDA. (Willing, F, 64 y.o.)*

*Prevention is better, and I believe in the wisdom of the MOH and the Mufti. (Willing, F, 29 y.o.)*

COVID-19 is not just a global pandemic and a public health crisis. The financial impact has already taken place worldwide. The sub-category of economic concern is the cue of action in taking the vaccines. Respondents believed that vaccination might reduce the number of COVID-19 infections. They believed that they could go back to their normal lifestyle that will eventually contribute to the country’s economic recovery and growth. Besides, some of them were concerned that the vaccines bought by the government would be wasted if it is not used. Therefore, they would instead take the vaccine than waste it.

*This is because vaccines are needed to ensure that the rate of infection is reduced to preserve economic and social conditions so that the economic downturn can be overcome and the development of the country can continue further. Thus the export-import process can be continued more effectively. (Willing, M, 56 y.o.)*

*Because it is already in our country, it means it has been bought, but the government needs to do a clinical test first… if the vaccine is not used at all, I worry that the country has wasted money in buying it. (Willing, F, 23 y.o.)*

The next cue for taking vaccine action and willingness to take the vaccines is that the respondents were not from the anti-vaccine group. In contrast, some respondents would prefer if the vaccines were administered to the high-risk categories.

*I am not anti-vaccine. (Willing, F, 41 y.o.)*

*I would expect those in the population most at risk to be given priority. We must give it to those high-risk categories first. (Willing, F, 45 y.o.)*

The final cue for action sub-categories is the wait-and-see behavior among respondents. Some of the respondents are unwilling to get vaccinated as they wanted to wait until more reliable data is available from those who had been vaccinated.

*Not confident with the research conducted. We should wait a little longer. (Not willing, F, 37 y.o.)*

*I will wait and see the reaction of others for any adverse effects. (Not willing, M, 64 y.o.)*

*I am willing when the vaccine is tested on humans for at least a year. (Not willing, F, 43 y.o.)*

## Discussion

This study identified several factors that could facilitate the introduction of COVID-19 vaccines using the Health Belief Model (HBM) as the theoretical framework for the community. Researchers were able to identify sub-themes related to participants’ acceptance of the vaccines. The sub-themes identified were: lack of awareness of perceived susceptibility, perceived severity, lack of self-efficacy, barriers and benefits of receiving the vaccine and their cue for action. The findings suggest that communities placed a high value on vaccines in general and were optimistic about the idea of a COVID-19 vaccine, with the majority willing to get vaccinated once it is available. This positive attitude is the general trend seen in another study conducted by Wong et al., 2020, which reported that 94.3% of participants in Malaysia responded positively to COVID-19 vaccine intent [21].

For the perceived susceptibility, knowledge and perception of vaccination were the influencing factors for vaccine acceptance. The respondents believed in the concept of ’prevention is better than cure’. This was also in line with previous studies that reported that individuals with better knowledge of diseases had higher practice scores [22, 23]. Thus, by getting vaccinated, they will protect themselves and their families from the risk of getting the SARS-CoV-2 infection. The Malaysian government has been actively conducting mainstream and social media campaigns on the COVID-19 vaccination programs to achieve herd immunity [24]. Herd immunity can be characterized as the indirect protection provided to susceptible individuals against infection when there is a sufficiently large proportion of immune individuals in a population [25]. Thus, many respondents mentioned their belief that herd immunity can break the chain of infection in the community. This is supported by the respondents’ responses on the success of the existing national immunization program provided free in all Ministry of Health facilities to Malaysian children [26].

Nevertheless, as knowledge was associated with their acceptance to get vaccinated, individuals who had a negative perception of COVID-19 were unwilling to get vaccinated as they did not acknowledge the existence of the COVID-19. This negative belief concurs with Jordan’s study, in which 45.1%, 45.0%, and 15.7% of respondents believed that COVID-19 disease is a punishment from God, a virus engineered in the laboratory, and bacteria, respectively cause it [27]. Interestingly, we found that the reason why a respondent was unwilling to get vaccinated was that they believed that COVID-19 was just propaganda.

For the perceived severity of the COVID-19 disease, community members also acknowledged that COVID-19 infection was a worrying condition, especially among the elderly, and they showed a positive attitude towards vaccination. It was also reported that the COVID-19 vaccine was highly accepted by the elderly in Indonesia and China [6, 28]. Consistent with previous studies, front-liners, including healthcare workers, frequent travelers and those with comorbidities, perceived that they were highly susceptible to COVID-19 due to the nature of their work and their health status, thus they were willing to get vaccinated [10, 11, 28]. However, the potential side effects were the influencing factor for the hesitation to accept vaccination. Since the COVID-19 vaccine was new, rapidly produced, had limited clinical trial data and unknown side effects, they were reluctant to get vaccinated. As this perceived health risk influences the individual’s decision to get vaccinated, which depends on the intensity and severity of side effects COVID-19 virus during the clinical trial, this data shall be transparent and available to the public [29].

The preventive measures taken by the government to control the spread of the COVID-19 pandemic include restriction of movement or lock-down and introduction of new norms, limiting human freedom [30, 31]. Therefore, the respondents believed that they would have the ability to lead a normal life after vaccination. Nonetheless, this is not quite true for the COVID-19 pandemic. After vaccination, the public is advised to keep wearing masks and practice social distancing until most people are vaccinated, and the scientists know more about the effect of vaccination [32]. It has been proven that vaccines reduce the symptoms and severity of infection, but whether or not the vaccine can prevent transmission is still questionable [33].

Twelve sub-themes emerged from the perceived barrier to getting vaccinated. These factors were similar to previously reported studies that influence the respondents’ willingness to get vaccinated [34, 35]. The newly produced vaccines, lack of correct or reliable information about vaccines, types of vaccines, mutation and *halal* status, have become significant barriers to accepting vaccination. Some respondents did not have confidence in the mRNA vaccine and preferred a well-established vaccine type such as an inactivated vaccine. Limited information on the vaccines and the limited clinical trial data also added up the concern. The ability of SARS-CoV-2 to mutate was thought to reduce the effectiveness of the vaccine, thus making them reluctant to get vaccinated. A study among 18,514 sequences showed little diversity across SARS-CoV-2 genomes, with only 11 sites showing polymorphisms in more than 5% of sequences, suggesting that a single vaccine candidate should be efficacious against currently circulating strains [36].

The *halal* status of the vaccines was amongst the perceived barriers to vaccination. Porcine-based gelatin, unslaughtered beef products, and aborted fetal material considered impure *(Haram)* in Islam had been used in vaccine production [37]. Meanwhile, other vaccines such as viral vectors and inactivated vaccines may use animal or human cells for virus propagations or gelatin as the stabilizer. In Indonesia, religious leaders also believed a *halal* label was required for community acceptance and maintenance of trust in their government and leaders [38]. In Malaysia, the Special Committee of National Council for the National Council for the Islamic Religious Affairs of Malaysia has agreed that the COVID-19 vaccine is compulsory for high-risk groups and permissible for others [39]. Besides, strict processes and regulations are surrounding *halal* certification and introducing a new vaccine in Malaysia. Religious authorities, community leaders, and healthcare providers need to develop strategies to gain public confidence to introduce a new vaccine.

Another sub-theme that emerged from the perceived barrier was the negative news in global media and the stigma and skeptical agenda on COVID-19 vaccination. Previous studies show that false information circulated by mainstream news and social media not only instigated confusion, fear and panic but also contributed to the development of misconceptions, disturbing and stigmatizing responses to COVID-19 and the vaccines produced by pharmaceutical companies [40]. This issue should be handled wisely to ensure that the public receive correct information from trusted sources. For example, in collaboration with the United Kingdom government, the WHO has taken the initiative to raise awareness of misinformation on COVID-19 and encourage individuals to report false or misleading content online [41].

While COVID-19 is more contagious than previous coronavirus infections, such as severe acute respiratory syndrome (SARS) and the Middle East respiratory syndrome (MERS), the case fatality ratio has been relatively lower. COVID-19 with 2.4% compared to MERS with 35% and 9% for SARS [42]. The elderly and those having comorbidity diseases are the most vulnerable groups [43, 44]. Therefore, young and healthy respondents believed that their immune systems were good enough to fight the virus, thus refusing vaccination. This is in line with a study that reported that being in the younger age group is an independent factor associated with vaccine hesitancy. Also, rather than taking the risk of getting the vaccine’s side effects, respondents put their trust in the available treatment in treating the COVID-19 disease due to the high recovery rate. Instead, they prefer to follow the government’s current regulations and standard operating procedures (SOP). A study in Hong Kong showed that willingness to accept the COVID-19 vaccine was lower in the third wave (34.8%) than in the first wave (44.2%). The factor contributed to decreasing willingness to accept the COVID-19 vaccine may be linked to increasing concerns about vaccine safety, the rush of vaccine development with limited clinical trial data and growing compliance of primary prevention [45].

Concerning the cost of COVID-19 vaccines, there were mixed responses to this issue. For some respondents, the willingness to get vaccinated relied on the vaccine’s cost. Several studies have been conducted on the willingness to pay (WTP) for COVID-19 vaccines. Being a healthcare worker, having a high income, and having high perceived risk were associated with higher WTP in a study conducted in Indonesia [46]. Similarly, in research conducted in Ecuador, WTP for the vaccine was associated with income, employment status, the perceived probability of needing hospitalization if contracting the virus causing COVID-19, and region of residence [47]. In Malaysia, the government promised that COVID-19 vaccines would be available for free with no out-of-pocket costs to all Malaysian residents [48]. Thus, other factors shall be considered during immunization programs, including the logistics and the accessibility for all to get vaccinated [26].

Under the HBM theme of cue for action concepts, six sub-themes emerged to take COVID-19 vaccines. For many respondents, acceptance was due to their desire as the vaccine will give protection. According to the respondents, it is their responsibility to protect themselves and their society against the virus. Once they get vaccinated, they can help in achieving herd immunity. This behavioral factor was a new factor that emerged from this study as most of the previous surveys only reported from individual and external factors [10, 35]. Additionally, as the COVID-19 pandemic has had a substantial negative impact on individuals and the country, many are willing to get vaccinated as they believe that once a country can stop the pandemic, the economy will be able to regain [49].

For those who were unwilling to get vaccinated, their cue relates to wait-and-see behavior. As it has only been less than a year since this newly developed vaccine was produced, the process of active decision-making takes time, as respondents seek out additional resources and mull over their decision. They want to wait and observe any negative consequences among vaccine recipients before committing themselves to vaccination. This behavior was similar to that observed in other studies when new vaccines were introduced to the community [50, 51].

Overall, the use of HBM in the present study can be considered an educational and behavioural intervention in the community to increase the acceptance of COVID-19 vaccination. A previous study that used the HBM in determining the coronavirus infection risk concluded that the HBM model could serve as a domain for communication processes and public health education [52]. While in another study conducted among the general population also suggests that educational intervention based on HBM can be considered a framework for the correction of beliefs and adherence to COVID-19 behaviour [53]. Thus in our study, by using the thematic analysis with HBM to analyse the qualitative response of participants, we confirmed the main factors contributing to the vaccine acceptance. Additionally, allowing the open-ended response allows the respondents to express their perceptions of the reasons behind their decision on the willingness or not willingness to get vaccinated. The study suggested that among those who are not willing to get vaccinated, the health information, education, and behaviour campaigns need to emphasise the benefits of vaccination and provide information to overcome the concern and barriers to getting vaccinated.

### Strength and limitations

This study provides an early insight into the COVID-19 vaccine’s acceptability in Malaysia using a qualitative approach. Despite all the challenges that COVID-19 has presented, there are many qualitative research opportunities. First, as well known in the literature, qualitative research deals with complex issues [54–56]. The study quality was enhanced by using open-text responses to develop insight into factors underpinning previously quantitative research in this area. Our study took place at the substantial peak of the COVID-19 pandemic in Malaysia before the second control movement order took place on 13 January 2021. Therefore, a longitudinal study to measure a COVID-19 vaccine’s acceptability at different intervals should also be conducted.

While robust, our study may have limitations that may affect the interpretation of our findings. Even though we have tried for a diverse set of respondents across Malaysia, because of snowballing methods used, there could be selection bias among the respondents that may over or under-estimate the different factors mentioned as motivators for vaccine acceptance.

This study also achieved a large sample size for in-depth research, which is adequate to use the HBM concepts as determinants of vaccine acceptance. Nevertheless, it was impossible to explore these variables’ views further when looking at open-text responses as the survey was conducted using Google Form.

### Implications for practice

As the vaccine development process continues, it will be essential to monitor the population’s vaccine acceptance. Our results highlight that vaccine acceptability may differ, influencing the people’s action of getting vaccinated. Healthcare providers’ essential role and trust, and modifiable health beliefs play a significant role in accepting COVID-19 vaccines. These findings can help guide the planning and development of future public health efforts to increase the acceptability and uptake of the COVID-19 vaccine.

Even though this study found that most responses showed a willingness to receive a COVID-19 vaccine when available, the government should take more action to increase the acceptance rate. This includes the need to build on generally positive community vaccine information, the availability of well-established vaccine delivery programs, increase and improve vaccine communication, and identify flexible, active health communication structures and information dissemination processes, thereby increasing the acceptance among the communities.

## Conclusion

The success of the COVID-19 vaccination program will rely heavily on public willingness to accept the vaccine once it is available. The health belief model in this study can be used to develop an intervention program to promote awareness and acceptance of the COVID-19 vaccine among the public in Malaysia. The public should be well informed about the vaccine, its efficacy, protection, and, most importantly, the *halal* status in a Muslim country to increase vaccine acceptability and achieve herd immunity. The refusal factor in getting vaccination was less likely due to the price factor but due to uncertainties of vaccine efficacy and *halal* status, lack of confidence in newly produced vaccines and the strong belief that their own body produces adequate immune protection. Therefore, of utmost importance, efforts must be made to understand and address factors that may affect COVID-19 vaccine uptake by all stakeholders, including medical experts and religious leaders.

## Abbreviations

COVID-19: Coronavirus disease 2019
F: Female
HBM: Health Belief Model
M: Male
SD: Standard deviation
y.o.: year old
